# Clinical Application of a New SARS-CoV-2 Antigen Detection Kit (Colloidal Gold) in the Detection of COVID-19

**DOI:** 10.3390/diagnostics11060995

**Published:** 2021-05-30

**Authors:** Evangelos Terpos, Ioannis Ntanasis-Stathopoulos, Miha Skvarč

**Affiliations:** 1 Department of Clinical Therapeutics, School of Medicine, National and Kapodistrian University of Athens, Alexandra General Hospital, PS 11528 Athens, Greece; johnntanasis@med.uoa.gr; 2 Clinical Trial Institution, General Hospital Jesenice, PS 4270 Jesenice, Slovenia; mihaskvarc@hotmail.com

**Keywords:** SARS-CoV-2, COVID-19, antigen test, nucleocapsid, clinical validation, nasal swab, nasopharyngeal swab

## Abstract

The precise diagnosis of COVID-19 is of outmost importance in order to effectively treat patients and prevent SARS-CoV-2 transmission. Herein, we evaluated the sensitivity and specificity of the COVID-19 Antigen Detection Kit (Colloidal Gold—CG) compared with PCR in nasopharyngeal and nasal samples. A total of 114 positive and 244 negative nasopharyngeal specimens confirmed by PCR were used in this comparative study. When the PCR positive Cycle Threshold (Ct) value was ≤25, CG sensitivity was 100%. When the PCR positive Ct value was ≤33, CG sensitivity was 99%. When the PCR positive Ct value was ≤40, CG sensitivity was 89.47%. Regarding nasal swabs, a total of 109 positive and 250 negative specimens confirmed by PCR were used. When the PCR positive Ct value was ≤25, CG sensitivity was 100%. When the PCR positive Ct value was ≤33, CG sensitivity was 96.12%. When the PCR positive Ct value was ≤37, CG sensitivity was 91.74%. Specificity was above 99% regardless of the Ct value of PCR positivity for both nasopharyngeal and nasal specimens. Overall, the CG showed high sensitivity and specificity when the PCR Ct value was less than 33. Therefore, CG can be used for screening early in the disease course. Confirmatory PCR is essential when a false negative result is suspected.

## 1. Introduction

The outbreak of the new coronavirus (SARS-CoV-2) infection has become a global public health crisis [1]. Research results show that the new coronavirus has a homology of 88 percent with the bat severe acute respiratory syndrome-like coronavirus (bat-SL-CoVZC45) [2,3]. Therefore, the Coronavirus Research Group of the International Commission on Classification of Viruses named the new pathogen “severe acute respiratory syndrome coronavirus 2” (SARS-CoV-2), and the disease was officially named as the corona virus disease 2019 (COVID-19) by the World Health Organization (WHO) [4].

SARS-CoV-2 is a single-stranded RNA virus that can primarily cause upper and lower respiratory tract infections after invading the human body. The main symptoms are fever, fatigue, and dry cough. In severe cases, breathing difficulties may gradually occur. Some patients have mild or even no symptoms, whereas others may present with a constellation of symptoms originating from several organs in the body [5]. Asymptomatic individuals do not present with any COVID-19-related symptoms, but as carriers of SARS-CoV-2, they may transmit the virus to other susceptible individuals. The condition of some critically ill patients may progress rapidly, and may lead to acute respiratory distress syndrome, septic shock, metabolic disturbances, hypercoagulation and multiorgan failure [5,6,7]. According to current epidemiological studies, the incubation period of SARS-CoV-2 is 1 to 14 days, usually 3 to 7 days. COVID-19 may be asymptomatic; however, asymptomatic carriers of SARS-CoV-2 may transmit the virus to vulnerable individuals [4].

Although the therapeutic approach for patients with COVID-19 has been significantly improved since the onset of the pandemic, prompt diagnosis remains the cornerstone of optimal therapeutic intervention and prevention of SARS-CoV-2 transmission [8]. The diagnosis of SARS-CoV-2 infection may be challenging depending on the clinical setting. The gold standard is the reverse-transcriptase polymerase chain reaction (RT-PCR); however, it may take a relatively long time (up to 24 h) for the result depending on the setting; it requires specialized personnel, and must be performed in laboratories of at least a P2 level, whereas its specificity is not 100% [9,10,11]. MALDI-TOF mass spectrometry may be superior to RT-PCR for SARS-CoV-2 detection, but it is not widely available [12]. Although most general hospitals have been now well equipped for performing PCR with the appropriate microbiological precautions, hospitals in rural areas or in low-resource settings and several clinics may lack the equipment and the personnel for safe and rapid PCR testing. Furthermore, the increased need of mass testing may cause important delays [11].

As an auxiliary diagnostic method, the antibody detection kit can simultaneously detect IgM and IgG antibodies [13]. The procedure is simple and convenient. It provides indirect evidence of the existence of the virus. It is mainly used for supplementary testing or joint testing of suspected patients with negative nucleic acid test results [14]. However, the production of antibodies requires a certain incubation period, which cannot be detected in patients in the window period. It is more suitable for patients two weeks or more after the onset of symptoms [15]. Those infected during the incubation period cannot be detected promptly, and may be able to transmit the virus. In addition to the above, the antibodies will last in the human body for a long time even after the patient is cured, antibodies may become evident following previous vaccination with anti-SARS-CoV-2 vaccines, and antibodies may show variations among individuals depending on the disease severity, age, sex and underlying medical conditions [16,17,18,19,20,21,22]. All these make difficult discrimination among current infection, past infection and prior vaccination, especially in cases of asymptomatic COVID-19 [23].

Furthermore, antigen detection kits aim to detect the pathogen itself, and, therefore, they provide direct evidence of viral infection even at the early stages of the disease [11,24]. The specimens of the antigen detection kit could be nasopharyngeal swabs, nasal swabs, oropharyngeal swabs and saliva samples. Nasal swabs, oropharyngeal swabs and saliva specimen can be relatively widely used in the clinical screening, and can be extended even for home self-testing.

In this context, herein we evaluated the COVID-19 Antigen Detection Kit (Colloidal Gold) produced by Zhuhai Lituo Biotechnology Co., Ltd., which aims to detect the N protein of the SARS-CoV-2.

## 2. Materials and Methods

### 2.1. Patient Selection

Two retrospective studies were designed to assess the COVID-19 antigen detection kit (colloidal gold) manufactured by Zhuhai Lituo Biotechnology Co., Ltd., Changsha, Hunan, China. The institutional review board of the General Hospital Jesenice approved the study (Number 2020/02). Participants provided oral informed consent for their samples to be evaluated for SARS-CoV-2 testing. We compared the results from the COVID-19 antigen detection kit (colloidal gold) with the RT-PCR results of samples from clinical cases with suspected COVID-19 in order to determine the sensitivity, specificity, and accuracy of the new method. As we aimed to assess the role of the COVID-19 antigen detection kit (colloidal gold) in the early detection of COVID-19, only patients reporting symptoms for a maximum of 7 days were included. Two kinds of specimens were used for evaluation: nasal swabs and nasopharyngeal swabs. Nasopharyngeal swabs are the most widely used clinical diagnostic specimens for the antigen test due to their high sensitivity and specificity [25]. However, nasopharyngeal swabs require an invasive procedure for collection performed, ideally, by a healthcare professional. In this context, nasal swabs are also accepted, especially for population screening, traveling controls and self-testing.

### 2.2. Sample Collection

All samples were collected from patients suspected of having COVID-19 at a single center in Slovenia. Nasal and nasopharyngeal swabs were simultaneously collected from the same patient. The swab specimens were collected as follows: The sampling swab was inserted into the nasopharyngeal/nasal cavity with the most secretions. The swabs were rotated gently, pressed on the nasopharyngeal/nasal wall three times, then inserted into the other nostril and the above steps were repeated. The swab head was then removed from the nasopharyngeal/nasal cavity quickly and was immersed in the sample treatment solution. After collection, the specimens were processed with the extraction buffer provided by the COVID-19 antigen detection kit (colloidal gold). The test was completed within 10 min. For negative specimens, the reagents actually can give out results in around 3 min. However, we still recommend to read the results between 10 to 15 min. Results after 15 min should not be considered valid. Left over fluids with appropriate swabs were transferred in two UTM mini transport media (UTM mini, Copan, Italy). Two specimens were used for extraction of RNA. Two specimens of each patient were collected simultaneously; one for the experiment kit and one for RT-PCR reagent detection. The CE marked two gene reverse transcriptase polymerase chain reaction (RT-PCR) (Seegene, South Korea) was used as comparator throughout the clinical evaluation. The handling of all samples was performed in accordance with the institutional rules of microbiologic safety.

### 2.3. Principles of Antigen Detection with the COVID-19 Antigen Detection Kit (Colloidal Gold)

The new coronavirus particles are wrapped with a lipid bilayer membrane. There are three glycoproteins on the membrane surface: Spike Protein (S, Spike Protein, which is the cell receptor binding site, the fusion site with the cell membrane); Membrane glycoprotein (E, Envelope Protein, a small protein that binds to the envelope); Membrane Protein (M, Membrane Protein, responsible for the transportation of nutrients across the membrane, the budding release of new viruses, and the formation of the virus envelope) [26]. One of the most important proteins in the viral nucleocapsid is the nucleocapsid protein (N, Nucleocapsid Protein), which is mainly responsible for the replication function of RNA. N protein is abundant in coronaviruses and is a highly immunogenic protein that participates in genome replication and cell signaling pathway regulation [26]. Therefore, Nucleocapsid protein (N protein) is often used as a diagnostic test tool for coronavirus, and is the core material of rapid immunological diagnostic reagents.

This kit adopts the double antibody sandwich method to qualitatively detect novel coronavirus antigens in human nasopharyngeal, oropharyngeal, and nasal swab specimens, and saliva specimens. The kit uses colloidal gold to label anti-human coronavirus monoclonal antibody 1. Then, coronavirus monoclonal antibody 2 and polyclonal antibody goat anti-mouse IgG is coated in nitrocellulose membrane.

When the tested sample is positive, the antigen in the specimen binds to the antibody 1 labeled with colloidal gold. Then, the antigen is chromatographed to the detection area and binds to the pre-coated anti-human coronavirus monoclonal antibody 2 to form a double antibody sandwich complex, which generates red color. Both antibody 1 and antibody 2 form specific binding with the N-protein on the SARS-CoV-2. The remaining colloidal gold-labeled antibody is combined with the polyclonal antibody at the quality control line to generate red color. Negative samples generate red color only at the quality control line.

The Cycle Threshold (Ct) value cut-offs of 25 and 33 are based on the normal requirements in most of the tenders in the EU. The Ct value of 40 can distinguish between the presence or absence of coronavirus infections in RT-PCR reagents; therefore, we set this cut-off as the highest value.

### 2.4. Statistical Analysis

The evaluation indicators for this clinical evaluation include sensitivity, specificity, and accuracy. The sensitivity of a test is its ability to determine the patient cases correctly. To estimate the sensitivity of the COVID-19 antigen detection kit (colloidal gold), we calculated the proportion of true positives in patient cases (PCR positive). The specificity of a test is its ability to determine the healthy cases correctly (PCR negative and no symptoms of COVID-19). To estimate the specificity of the COVID-19 antigen detection kit (colloidal gold), we calculated the proportion of true negative in healthy cases. The accuracy of a test is its ability to differentiate the patient and healthy cases correctly. To estimate the accuracy of the COVID-19 antigen detection kit (colloidal gold), we calculated the proportion of true positive and true negative in all evaluated cases. We used Cohen’s Kappa formula to analyze the raw data.

## 3. Results

Overall, 358 nasal and 358 nasopharyngeal swabs were simultaneously collected from the same patients. One additional nasal swab was taken from a patient that developed another symptom of COVID-19 the next day.

### 3.1. Nasopharyngeal Swabs

Regarding the evaluation of nasopharyngeal samples, the COVID-19 antigen detection kit (colloidal gold) and PCR detection kit were used simultaneously to test nasopharyngeal swab specimens, which were sampled at the Primary Care Health Care institution and General Hospital Jesenice, Slovenia. A total of 114 positive and 244 negative specimens confirmed by PCR were used in this comparative study. The minimum Ct value of PCR positivity was 13.4 and the maximum Ct value was 38.4. There were 70 specimens with a Ct value of less than 25, 30 specimens with a Ct value between 25 to 33, and 14 specimens with a Ct value above 33.

When the Ct value of PCR positivity was less than 25, the antigenic test detected all 70 PCR positive samples. The antigenic test detected only one false positive out of 244 PCR negative samples (Table 1). Therefore, the detection sensitivity was 100% (95% CI: 98.79–100.00%) and the specificity was 99.59% (95% CI: 98.07–99.91%) for PCR Ct values less than 25. Furthermore, encouraging results were found in Ct values of PCR positivity between 25 and 33. Only one PCR positive specimen was not detected by the antigen test and only one PCR negative specimen was assigned as PCR positive by the antigen test. Therefore, the sensitivity of the antigen test was 99% (95% CI: 97.28–99.64%) and its specificity was 99.59% (95% CI: 97.28–99.64%) (Table 2). However, if the Ct value of PCR positivity rises beyond 33, the sensitivity of the antigen test decreases rapidly to 89%. Overall, there were 12 specimens with a Ct value between 33 and 38, whereas most of them had a Ct value above 35. The overall agreement between RT-PCR and the COVID-19 antigenic test was still high and reached 96.37%; however, the sensitivity decreased to 89.47% (Table 3).

### 3.2. Nasal Swabs

Regarding the evaluation of nasal swabs, we performed a comparative study between the new antigen test and RT-PCR. The COVID-19 antigen detection kit (colloidal gold) and PCR detection kit were used simultaneously. A total of 109 positive and 250 negative specimens confirmed by PCR were used in this study. The Ct value of PCR positivity ranged from 16.7 to 36.9. Among the PCR positive samples, 61 had a Ct value less than 25, 103 had a Ct value less than 33, and 109 had a Ct value less than 37.

Among the samples with PCR Ct values less than 25, the antigen test detected all positive specimens (Table 4). Importantly, all 250 PCR negative specimens were assigned as negative by the antigen test as well (Table 4). Therefore, the sensitivity and specificity of the antigen test were both 100%, respectively. When the Ct value of PCR positivity increased to 33, the antigen test detected 99 out of 103 PCR positive samples as true positives (Table 5). Therefore, the sensitivity of the antigen test reached 96.12% (95% CI: 90.43–98.49%). For Ct values of PCR positivity below 37, there were 100 true positive and 9 false negative specimens according to the antigen test, among 109 PCR positive samples (Table 6). Therefore, the sensitivity of the antigen test was 91.74% (95% CI: 85.05–95.60%).

## 4. Discussion

In this study we present an independent clinical evaluation of the immunochromatographic COVID-19 antigen detection kit (colloidal gold) in nasopharyngeal and nasal swabs compared with RT-PCR, among patients suspected of having COVID-19. The new antigen test had excellent diagnostic accuracy (above 95%), regardless the type of specimen and the Ct value of PCR positivity. The specificity of the antigen test was also above 99% in all settings. The sensitivity of the antigen test was really encouraging and ranged between 89% and 100%. When the Ct value of PCR positivity increased to 33 and above, a drop in sensitivity became evident in both the nasopharyngeal and nasal specimens. Higher Ct values of PCR positivity are associated with a decreased concentration of SARS-CoV-2 viral particles in the solution and, therefore, the detection rate of positive samples with the antigen test decreases. For this reason, we included only patients suspected of having COVID-19 and with suspected COVID-19-related symptoms with a duration of no more than 7 days, in order to assure an adequate concentration of the virus in the specimens.

Furthermore, the absolute sensitivity value was lower for the nasal specimens in Ct values of PCR positivity less than 33 but higher for the nasal specimens in Ct values of PCR positivity less than 40, as compared with the corresponding values for the nasopharyngeal specimens. These slight differences may be attributed to the number of patients with high PCR Ct values in each sub study; more specifically, 8 patients had PCR Ct values between 35 and 38 in the nasopharyngeal sub study, whereas in the nasal sub study only 2 patients had PCR Ct values in that range.

Our results are comparable to other studies. One study compared two analyzer-based tests to single gene SARS-CoV-2 RT-PCR. The chromatographic immunoassay BD Veritor System for Rapid Detection of SARS-CoV-2 nucleocapsid antigen (Veritor) showed a high positive percent agreement with the PCR results ranging from 81.8% to 87.5%, which is compatible to the high accuracy rates found in our study [27]. Based on these encouraging results, the FDA provided an emergency use authorization for Veritor as a point-of-care test for patients suspected of having COVID-19 presenting in less than 7 days from symptom onset.

Another study compared several immunochromatographic antigen tests to RT-PCR. Sensitivity was between 16% to 85%. The wide sensitivity range was attributed to the use of non-validated samples [28]. In one study the antigen test identified 70.6% of RT-PCR positive samples. A major limitation of this study was that the authors diluted the sample in transport media and performed the analysis off-site [29]. In comparison, our study was performed on fresh samples as a point-of-care test. This approach may explain our superior results compared to other studies using stored frozen samples to validate their antigen test in an offsite setting.

Another study evaluated the Standard Q COVID-19 Ag test (SD Biosensor, Chuncheongbuk-do, Republic of Korea), which is a rapid chromatographic immunoassay, for the detection of SARS-CoV-2 nucleocapsid antigen in 454 respiratory specimens. Similar to our results, the sensitivity and specificity were 98.33% and 98.73%, respectively [30].

FindDx platform has published several validations of immunochromatographic antigen SARS-CoV-2 assays. Our approach showed similar performance to the best SARS-CoV-2 antigen immunochromatographic tests in other comparisons (sensitivity between 76.6% and 90.8%, specificity between 99.3% to 100%) [31,32]. A study that used an analyzer to screen a student population resulted in inferior sensitivity compared to our study [31]. Importantly, our results with the colloidal gold assay are in accordance with the manufacturer performance data, which demonstrated 96.9% sensitivity and 99.5% specificity [31]. The drop in sensitivity of our evaluation can be explained by the low viral load in patients that did not present early during the course of COVID-19.

In a Cochrane meta-analysis, the sensitivity of antigen tests varied considerably from 0% to 94% across five studies on 943 samples [33]. In an updated version of the meta-analysis, 48 studies encompassing 58 evaluations of antigen tests were included. Interestingly, the average sensitivity was higher in the first week after symptoms onset (78.3%, 95% CI: 71.1–84.1%) compared with the second week of symptoms (51.0%, 95% CI: 40.8–61.0%). Sensitivity was high among those with PCR Ct values less than 25 (94.5%, 95% CI: 91.0–96.7%) compared with those with Ct values more than 25 (40.7%, 95% CI: 31.8–50.3%) [24]. These results are in accordance with our study, which shows superior sensitivity of the new antigen test in the early period from the onset of COVID-19 related symptoms, and among positive samples with a lower threshold of PCR positivity.

Our study has some limitations. It was performed in a single centre and, thus, the level of expertise of the personnel performing the sampling may be different from other hospital settings. Therefore, the education of all health care workers is very important in order to safely and accurately perform this procedure. Furthermore, we included only symptomatic patients presenting with symptoms with a duration of less than 7 days; thus, generalization of our results for asymptomatic carriers and patients with long lasting symptoms should be made with caution. Another potential limitation lies in the fact that our study focuses on RT-PCR for the diagnosis of COVID-19. Although the approved RT-PCR detection kits have high sensitivity and specificity rates, the results should be interpreted in association with clinical and radiological findings in a tailored manner.

In conclusion, our results show that the COVID-19 Antigen Detection Kit (Colloidal Gold) fulfils the WHO criteria for point-of-care COVID-19 tests of 80% sensitivity and 97% specificity in health care settings. Nasopharyngeal and nasal swabs have comparable high sensitivity, specificity, and accuracy in symptomatic patients with COVID-19 symptoms experienced for less than 7 days. Nasal swabs may be more convenient to collect in the clinical practice, especially in high patient volume settings. In the case of a negative antigen test despite high COVID-19 suspicion, a confirmatory PCR is necessary as the gold standard method.

## Figures and Tables

**Table 1:** Test results and analysis of nasopharyngeal swab specimens with Ct values of PCR positivity less than 25.

*n*	Nasopharyngeal Swab Specimen		
	Positive (PCR < 25)	PCR Negative	Total
AT Positive	70	1	71
AT Negative	0	243	243
Total	70	244	314
Sensitivity	100.00%		
95% CI	98.79–100.00%		
Specificity	99.59%		
95% CI	98.07–99.91%		
Accuracy	99.68%		

AT: antigen test; CI: confidence interval.

**Table 2:** Test results and analysis of nasopharyngeal swab specimens with Ct values of PCR positivity less than 33.

*n*	Nasopharyngeal Swab Specimen		
	Positive (PCR < 33)	PCR Negative	Total
AT Positive	99	1	100
AT Negative	1	243	244
Total	100	244	344
Sensitivity	99.00%		
95% CI	97.28–99.64%		
Specificity	99.59%		
95% CI	98.18–99.91%		
Accuracy	99.42%		

AT: antigen test; CI: confidence interval.

**Table 3:** Test results and analysis of nasopharyngeal swab specimens with Ct values of PCR positivity less than 40.

*n*	Nasopharyngeal Swab Specimen		
	Positive (PCR < 40)	PCR Negative	Total
AT Positive	102	1	103
AT Negative	12	243	255
Total	114	244	358
Sensitivity	89.47%		
95% CI	85.86–92.24%		
Specificity	99.59%		
95% CI	98.22–99.91%		
Accuracy	96.37%		

AT: antigen test; CI: confidence interval.

**Table 4:** Test results and analysis of nasal swab specimens with Ct values of PCR positivity less than 25.

*n*	Nasal Swab Specimen		
	Positive (PCR < 25)	PCR Negative	Total
AT Positive	61	0	61
AT Negative	0	250	250
Total	61	250	311
Sensitivity	100.00%		
95% CI	94.08–100.00%		
Specificity	100%		
95% CI	98.49–100.00%		
Accuracy	98.78%		
95% CI	98.78–100.00%		

AT: antigen test; CI: confidence interval.

**Table 5:** Test results and analysis of nasal swab specimens with Ct values of PCR positivity less than 33.

*n*	Nasal Swab Specimen		
	Positive (PCR < 33)	PCR Negative	Total
AT Positive	99	0	99
AT Negative	4	250	254
Total	103	250	353
Sensitivity	96.12%		
95% CI	90.43–98.49%		
Specificity	100.00%		
95% CI	98.49–100.00%		
Accuracy	98.87%		
95% CI	97.12–99.56%		

AT: antigen test; CI: confidence interval.

**Table 6:** Test results and analysis of nasal swab specimens with Ct values of PCR positivity less than 37.

*n*	Nasal Swab Specimen		
	Positive (PCR < 37)	PCR Negative	Total
AT Positive	100	0	100
AT Negative	9	250	259
Total	109	250	359
Sensitivity	91.74%		
95% CI	85.05–95.60%		
Specificity	100.00%		
95% CI	98.49–100.00%		
Accuracy	97.49%		
95% CI	95.30–98.68%		

AT: antigen test; CI: confidence interval.

## Data Availability

Data available from the authors upon request.
